# Active learning applied to automated physical systems increases the rate of discovery

**DOI:** 10.1038/s41598-023-35257-7

**Published:** 2023-05-24

**Authors:** Michael D. Shields, Kurtis Gurley, Ryan Catarelli, Mohit Chauhan, Mariel Ojeda-Tuz, Forrest J. Masters

**Affiliations:** 1 Department of Civil and Systems Engineering, Johns Hopkins University, Baltimore, MD 21212 USA; 2 Department of Civil and Coastal Engineering, University of Florida, Gainesville, FL 32611 USA

**Keywords:** Engineering, Civil engineering

## Abstract

Active machine learning is widely used in computational studies where repeated numerical simulations can be conducted on high performance computers without human intervention. But translation of these active learning methods to physical systems has proven more difficult and the accelerated pace of discoveries aided by these methods remains as yet unrealized. Through the presentation of a general active learning framework and its application to large-scale boundary layer wind tunnel experiments, we demonstrate that the active learning framework used so successfully in computational studies is directly applicable to the investigation of physical experimental systems and the corresponding improvements in the rate of discovery can be transformative. We specifically show that, for our wind tunnel experiments, we are able to achieve in approximately 300 experiments a learning objective that would be impossible using traditional methods.

## Introduction

Active learning is a subfield of machine learning (ML) in which an algorithm uses previously collected data to identify the most informative computational or physical experiment to run next, thus optimizing the learning rate for a specified objective^1^. In the past 20 years, active learning has grown into a discipline much of its own, with particular emphasis on the development of novel learning functions and their coupling with different ML methods. Prior applications of active learning (e.g. for Bayesian optimization^2^) have largely focused on the design of computer experiments in which the ML algorithm identifies the precise parameter values with which to initialize a computer model. Very recently, some related approaches have employed reinforcement learning (often used for active control) for the design of computational experiments as well, e.g.^3–6^. Such applications are ubiquitous and are increasingly recognized as the state-of-the-art in design of computational experiments (see e.g.^7–9^).

In contrast to computational experiments, far fewer applications of active learning (or reinforcement learning^10^) for physical experiments can be found, largely due to the cost and logistical challenges associated with the automation of complex physical experiments. Several authors have composed “how to” articles that shed important light on potential future capabilities—for example in chemical^11–14^ and materials discovery^15–19^, physics^20^, and in the biological sciences^21–25^. In the wind engineering field specifically, where large-scale physical testing is often required and numerical simulations remain an insufficient replacement, a few studies have used ML-based predictors derived from computational models with Boundary Layer Wind Tunnel (BLWT) experiments to validate the results^26^ or to control active flow purely using a priori numerical simulations^27,28^. But across fields, the potential for transformative discoveries remains largely unrealized, and active learning enabled discoveries from physical experiments remain largely hypothetical.

**Figure 1:**
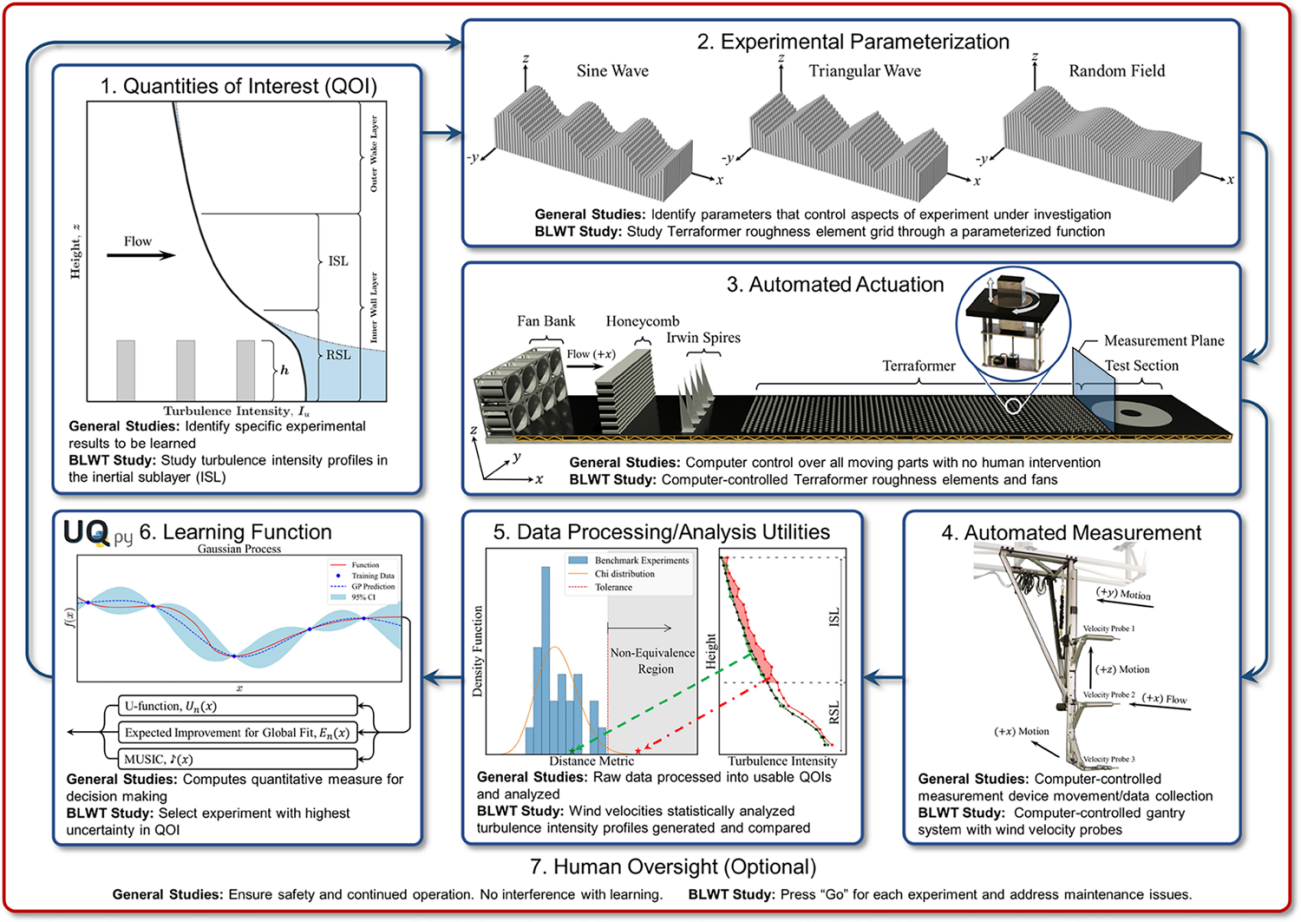
Schematic of the general active learning framework with specific application to boundary layer wind tunnel automation and learning.

In this work, we describe and employ a novel framework for active learning within large-scale physical systems and apply it using actively learned BLWT experiments to discover fundamental relationships between terrain roughness, the resulting near-surface atmospheric turbulence, and ultimately the wind pressures needed to design critical infrastructure (Fig. 1). Our automated and active learning-controlled experiments are enabled by the combination of effective learning functions, a novel automated “Terraformer” that rapidly modulates the tunnel’s aerodynamic surface roughness, and a mechanized instrument traverse to measure experimental outcomes. We demonstrate that active learning can offer orders of magnitude reductions in the number of experimental configurations needed to enable foundational discoveries that would be otherwise infeasible.

## Methods

### General active learning framework

Active learning for large-scale experimental investigations requires a tightly coordinated closed-loop system with active feedback between experimental equipment/instrumentation and data analysis/ML software. Our framework (Fig. 1) is comprised of the following essential components:

1. *Quantities of interest:* Each experiment must have a carefully selected and quantifiable objective in the form of a quantity of interest (QOI) or set of QOIs. The QOI is the measurable result of the experiment. It is a physically meaningful and informative takeaway from the experiment that serves as the basis for learning and discovery.
2. *Experimental parameterization:* The experimental design space must be appropriately parameterized and parameter ranges/distributions identified. An experimental design space may require a single parameter or hundreds of parameters. For expensive and time-consuming experiments, a down-selection mechanism is critical to identify the most influential parameters.
3. *Automated/controlled actuation:* Given a set of parameters, execution of the experiment must be automated. This requires a mechanized and controlled experimental apparatus that can configure and initialize experiments without requiring human intervention.
4. *Automated measurement instrumentation:* Instruments for measuring the data from which the QOI is extracted must be computer controlled and automated to eliminate costly and time-consuming manual operations, and coordinated and/or sequenced with automated experimental actuation.
5. *Data processing/analysis utilities:* Processing and/or analysis of the raw data from automated instrumentation is generally necessary. This may be simply extracting individual values (e.g., peaks) or performing complex regressions, optimization, or dimension reduction to extract salient features from the data. This data processing must be conducted rapidly “on-the-fly” to extract the QOIs used for discovery and the associated active learning to select the next experiment.
6. *Learning function:* The learning function is the decision-making algorithm that specifies the parameter values for the next experiment based on previous QOIs. The learning function is typically designed from some underlying ML algorithm, but may also result from statistical analysis, data-driven learning methods, or even physics-based computations. The learning function must have low computational cost to avoid delays.
7. *Human oversight (optional):* Many experiments, especially those using large-scale and/or potentially dangerous or hazardous equipment, require that a human operator supervise certain aspects of the process. This generally involves human oversight of automated systems, human intervention as a failsafe, or perhaps an individual to manually initialize or verify a potentially risky automated sequence. Importantly, this human does not aid the learning process or interfere with the automated experiment.

### Scope of the present study

We demonstrate how this active learning framework enables experimentally driven discoveries in a long-fetch open-circuit boundary layer wind tunnel (BLWT) having total dimensions of 6 m W $\times$ 3 m H $\times$ 38 m L. Boundary layer flow development is investigated using controlled actuation of an automated roughness element grid that generates mechanical turbulence.

In a BLWT, fan-driven air is forced through a long duct with an array of roughness elements mounted to the floor to grow a boundary layer (BL) along the length of the tunnel. Near the floor, turbulence is created via mechanical mixing as air flows around the roughness elements. This influence of the surface roughness on turbulent behavior decays with height, as characterized by a turbulence BL profile (Fig. 1—inlay 1.), and depends on the spacing, shape, orientation, and height of the roughness elements. A trial-and-error procedure is typically utilized by manually adjusting roughness element arrays to simulate properly scaled standard BL profiles associated with terrains such as ocean-front, open farmland, or suburban conditions. The University of Florida BLWT (Fig. 1—inlay 3.) replaces the manual roughness adjustment approach with the Terraformer - a unique computer-controlled array of 1116 individual roughness elements (62 rows of 18) with fetch length of 18.3m that rapidly actuate to user-specified configurations^29–31^. Catarelli et al.^29^ describes the characteristics, operation, and limitations of the system.

We harness the proposed active learning framework to discover families of Terraformer roughness grid configurations that produce statistically equivalent BL turbulence profiles. The goal is to reveal the critical relationship between terrain, turbulence and the resultant pressures on infrastructure being designed to resist wind loads. Statistical equivalence is defined through height dependent turbulence intensity (second-order) profiles of the wind velocity immediately downstream from the Terraformer roughness grid (Turbulence intensity is far from a complete descriptor of the turbulence profile. However, extensive investigations of various metrics, including complete power spectral profiles, demonstrated that turbulence intensity expresses many of the second-order salient features of the flow profile, while remaining scalar valued and thus easy to work with. Additional studies using more detailed descriptors are certainly possible, and this framework opens the door to such investigations). Any two profiles are considered statistically equivalent if the norm of their difference lies below a threshold, elaborated further below. The focus of this work is on the rapid discovery of second-order equivalent profiles enabled by active learning, while the significance of these second-order equivalent profiles to the engineering community will be presented in relevant domain-specific periodicals.

We apply the active learning framework to identify different Terraformer configurations that produce wind fields that are 2nd-order equivalent to those produced by a uniform grid with element heights all set to 80 mm (the benchmark configuration). This benchmark case for which equivalence is being sought creates mean and turbulence intensity profiles that match the standard models and metrics employed to investigate pressures on scale models of bluff body infrastructure (e.g. buildings)^29,30^, and are therefore of direct value to the wind engineering community. Each of the active learning components are described below with algorithmic and equipment design details provided in the Supplementary Materials (SM).

#### Quantities of interest

This investigation seeks to discover 2nd-order equivalence between wind velocity fields generated from different terrains that achieve a surface-roughness Reynolds number $Re_{*} = u_{*}z_{0}/\nu \ge$ 2.5—defined by Schlichting^32^ to be aerodynamically fully-rough—where $u_{*}$ is shear velocity, $z_{0}$ is aerodynamic roughness length, and $\nu$ is the kinematic viscosity of air . Our QOI measures the difference in the second-order statistical profiles of the wind velocity, defined through the turbulence intensity profile given by:

1 $$\begin{aligned} l_x(z) = \dfrac{\sigma (z)}{\mu (z)} \end{aligned}$$

where $\sigma (z)$ and $\mu (z)$ are the standard deviation and mean velocity of the wind in the along-wind (x) direction at height (z) above the floor. Specifically, our QOI is defined as the L2 distance between two turbulence intensity profiles

2 $$\begin{aligned} d = \left| \left| l_x^\theta (z) - l_x^b(z)\right| \right| _2 \end{aligned}$$

where $l_x^\theta (z)$ is the experimental profile having parameters $\theta$, and $l_x^b(z)$ is the benchmark profile averaged over 25 repeated benchmark experiments with all element heights at 80 mm. The criterion $d<\epsilon$ defines second-order equivalence where $\epsilon$ is determined as the $99\mathrm{th}$ percentile from a chi distribution obtained from statistical analysis of the 25 repeated benchmark experiments. See SM for a detailed derivation.

#### Experimental parameterization

The Terraformer can be characterized by as many as 2232 degrees of freedom (i.e. the height and rotation angle for each element). We parameterized these degrees of freedom by choosing functional forms describing shapes that traverse the 62 rows of elements in the along-wind direction (the x-axis in Fig. 1—inlay 2 and 3.). We investigate five versions of this concept in three phases. Phase 1 controls the amplitude and wavenumber of a single sine wave (the phase and mean value are fixed). Phase 2 controls the mean element height and amplitude of three different wave shapes—sine wave, triangular wave, and square wave. Phase 3 controls the parameters of a discretized random field (Fig. 1—inlay 2.). Details of these phases and the corresponding Terraformer parameterizations are provided in Table 1 with additional information in the SM.

**Table 1:** Description of the three phases of experiments conducted in this study, including experimental parameterization.

Phase	Terrain description	Functional form and parameters			Learning function
1.	Sine wave	$H(x)=\mu _H + A\sin (\omega (29,500-x)+\pi )$			Noisy U Noisy EIGF
		Mean element height $\mu _H$	Amplitude *A*	Wave number $\omega$	
		80 mm	*U*(0, 80) mm	$U(-\frac{2\pi }{3000}, \frac{2\pi }{3000})$ rad/mm	
2.	(a) Sine wave (b) Triangular wave (c) Square wave	$$\begin{array}{cc} &{}H_a(x)=\mu _H + A \sin (-\omega (29,500-x)) \\ &{}H_b(x) = \mu _H + \frac{2A}{\pi }\arcsin (\sin (-\omega (29,500-x))) \\ &{}H_c(x) = \mu _H + A \text { sgn}(\sin (-\omega (29,500-x)))\end{array}$$			Noisy U
		Mean element height $\mu _H$	Amplitude *A*	Wavenumber $\omega$	
		*U*(50, 110) mm	*U*(10, 30) mm	$-\frac{\pi }{3000}$ rad/mm	
3.	Random field	$H(x)=\mu _H + \sum _{i=1}^n \theta _i \sqrt{\lambda _i} f_i (29,500-x)$			MUSIC Noisy U
		Mean element height $\mu _H$		Random field amplitudes $\theta _i, i=1, \ldots , n$	
		80 mm		*N*(0, 1)	

Notes: (i) Learning function names refer to Table 2. (ii) Details of the Phase 3 expansion *H*(*x*) are found in the “Results” section below.

#### Automated measurement instrumentation

A mechanized instrument traverse in the BLWT is outfitted with three probes that measure the three fluctuating components of wind velocity (*x*, *y*, *z*) and static pressure in real-time (Fig. 1—inlay 4.). The probes collect data for 30 s and then move vertically in 20 mm increments to collect data at the next position. In total, data are collected over a height range from 180 to 500 mm in a plane perpendicular to the wind direction (Fig. 1—inlay 3.). The traverse and probe system automatically collect and transmit the necessary data to a computer to produce the QOI in the automated experimental sequence.

#### Data processing/analysis utilities

Data collected from the traverse-mounted probes are collected through a Labview DAQ system and stored to .th files that are read into a Matlab processing script that evaluates the turbulence intensity profile (1st QOI). Turbulence intensity profiles are then passed into a Python script that evaluates the distance from the benchmark profile (2nd QOI) and runs the learning function (described next) to identify the Terraformer parameter values for the next experiment. The script then generates the new Terraformer configuration file and notifies the equipment operator. In total, data processing and learning takes approximately 90 s per experiment.

#### Learning function

The automated BLWT active learning framework is driven by a learning function whose objective is to identify the most informative experiment to run given the results of all prior experiments. We explored three learning functions summarized in Table 2.

**Table 2:** Description of the learning functions employed in this study.

Learning function	Expression	Objective
Noisy U-function	$U_n(\theta )=\frac{\mu _g(\theta )}{\sqrt{\sigma _g^2(\theta )-\sigma _{\epsilon }^2(\theta )}}$	Goal: Conduct experiments along the parameter surface separating the 2$^{\mathrm{nd}}$ order equivalent and non-equivalent regions.
		How: Conduct experiments that have the highest probability of incorrectly predicting the sign of the performance function.
Noisy EIGF (Expected Improvement for Global Fit)	$E[I_n(\theta )]=\bigg ( \mu _g(\theta ) - g(\theta ^*)\bigg )^2 + \sigma _g^2(\theta ) - \sigma _{\epsilon }^2(\theta )$	Goal: Conduct experiments that globally best approximate the performance function.
		How: Conduct experiments that have both high prediction uncertainty and large difference from nearby experiments.
MUSIC		Goal: Conduct experiments that allow efficient computation of sensitivity indices.
		How: Conduct experiments that have both high prediction uncertainty and large differences from nearby experiments in conditional GP

Note: Definition of the terms in each equation can be found in the text body.

All learning functions are constructed from a Gaussian Process (GP) regression model^33^ used to predict the distance between the turbulence intensity profile of an as-yet untested parameterized Terraformer configuration and the benchmark turbulence intensity profile, along with a measure of the uncertainty in this prediction. More specifically, we define a performance function

3 $$\begin{aligned} g(\theta ) = d \left( l_x^\theta , l_x^b \right) -\epsilon = \left| \left| l_x^\theta (z) - l_x^b(z)\right| \right| _2 - \epsilon \end{aligned}$$

such that $g(\theta )\le 0$ corresponds to second-order equivalence and $g(\theta )>0$ corresponds to second-order different profiles, where $\theta$ are the Terraformer configuration parameters, $\epsilon$ is the threshold distance value for second-order equivalence. The GP regression model gives both a mean prediction $\mu _g(\theta )$ and the associated standard deviation $\sigma _g(\theta )$ as a measure of uncertainty in the prediction. The Noisy U-function and Noisy EIGF, modified from^34^ and^35^ respectively to include noise in the GP, utilize these terms directly. Meanwhile, the MUSIC (Minimizing Uncertainty in Sensitivity Index Convergence) uses a main effect GP defined by the conditional expectation $A(\theta ^{(i)})=E_{\theta ^{(-i)}}\left[ g|\theta ^{(i)}\right]$ where $\theta ^{(i)}$ represents the $i\mathrm{th}$ component of $\theta$ and $\theta ^{(-i)}$ denotes all components of $\theta$ except component *i*, which has mean $\mu _{A^{(i)}}(\theta ^{(i)})$ and standard deviation $\sigma _{A^{(i)}}\left( \theta ^{(i)}\right)$^36^.

#### Human oversight

The wind tunnel operator initializes the fans to the corresponding RPMs and, once the fans have reached the steady state the operator is in charge of running the LabVIEW interface to start collecting the data. Once the data collection is finished per experiment, the operator executes the Python script mentioned in the “Data processing/analysis utilities” section to generate the next Terraformer configuration. The input file of the new Terraformer configuration is then loaded into the BLWT computer, and the process starts again. The safety-driven human operator actions account for less than 2 min of the 20 min needed per experiment, and the operator never interferes with the learning process.

## Results

We studied the efficacy of the active learning approach in three phases of experiments (Table 1). The first phase served as a proof of concept, the second was a validation exercise, and the third was a more comprehensive study of higher complexity intended to push the limits of active learning enabled discovery. Prior to any of the three heterogeneous element array phases described in Table 1, we completed 25 identical uniform element array experiments (every element set to 80 mm) to provide the benchmark frame of reference for 2nd order equivalence exploration. The turbulence intensity profile averaged through these 25 experiments at each height provided the target second order profile, while the 25 individual turbulence intensity profiles provided the means to quantify the uncertainty among identical benchmark experiments at each height (See SM). Second order equivalence thus refers to whether the turbulence intensity profile from any individual heterogeneous element array experiment (from any of the 3 phases) is statistically ‘within the experimental noise’ of the benchmark turbulence intensity profile.

### Phase 1: Sine wave configuration & proof of concept

In phase 1, we considered a simple one-dimensional sine wave Terraformer element configuration in the along-wind direction with two variable parameters, the sine wave amplitude and the wave number (Table 1), with the mean and phase fixed. The study served as a proof of concept, allowing us to “learn the learning” by applying two different learning functions to explore their capacity to achieve our objective of identifying second-order equivalence within the defined parameter space.

We began by conducting 25 experiments with different sine wave Terraformer configurations to train the GP surrogate and initialize the learning. We then performed 120 active learning experiments using the noisy U-function in which the amplitude was constrained by the maximum/minimum allowable element heights (160 mm and 0 mm, respectively), five manual experiments for validation (no learning), followed by 78 additional experiments over a reduced parameter space that focused on the region of second order equivalence with amplitude $A\in [0, 30]$ mm using the noisy EIGF. In total, 253 total experiments were conducted over 96 non-sequential hours. Despite using a variety of learning methods, and some manual intervention, this initial exploration of the learning gave us sufficient confidence that we could efficiently identify regions in the parameter space that resulted in second-order equivalence using the proposed learning function to select subsequent experiments.

### Phase 2: Wave shape study

**Figure 2:**
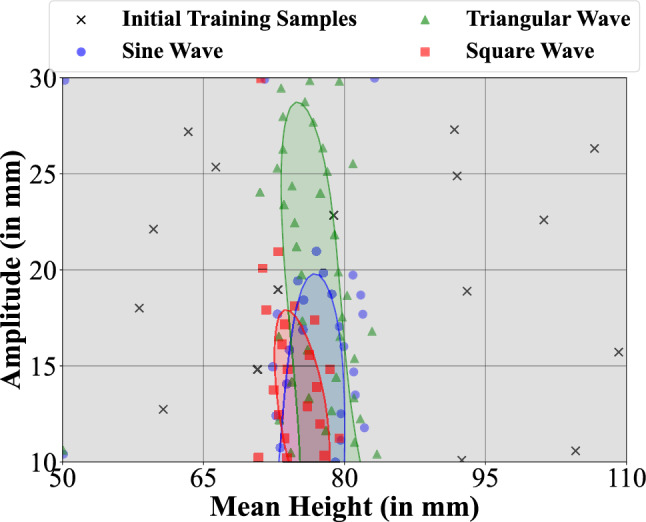
Phase 2 shape study—regions of 2nd order equivalance for sine wave (blue), triangular wave (green), and square wave (red) terrains bounded by the limits of the experimental amplitude [10, 30] mm and mean height [50, 110] mm.

In phase 2, we investigated the influence of three simple wave shapes (sinusoidal, triangular, and square) as a means of demonstrating the learning and its ability to identify the region of 2nd-order equivalence for related low-dimensional parameterized surface roughness. The three wave shapes were parameterized identically, varying the mean element height and wave amplitude over a range of interest identified from Phase 1 (Table 1). In particular, the minimum amplitude of 10 mm ensured that all experiments would be sufficiently different from the benchmark (having zero amplitude) while extending to sufficiently large mean heights and amplitudes to capture the limits of equivalence. We were interested in studying these differences in shape to better understand the influence of different terrain features on turbulence profiles and learning. We initialized the learning by running 16 initial experiments with the same parameters, selected using stratified sampling to cover the space, for each shape to train the GP surrogate. We then ran active learning with the noisy U-function for a total of 43, 56 and 37 experiments for sine, triangular and square waves, respectively, over 78 h to adequately resolve the regions of 2nd-order equivalence. These regions are shown in Fig. 2, where the solid lines showing the boundaries of the predicted equivalence regions are identified as the surface corresponding to $g(\theta )=0$ from Eq. (3) using the actively learned GP surrogate. Observe that the sine and square wave profiles have very similar equivalence regions although the square wave region extends over a slightly narrower range of wave numbers and slightly lower amplitudes. This is perhaps intuitive because the square profiles have sharper transitions that will cause added turbulence. The second-order region for triangular waves, on the other hand, extends over similar wave numbers but extends to much higher amplitudes (up to $\sim$ 1 cm higher). This is because fewer elements project higher into the flow and the transitions are gradual, causing less turbulence. Importantly, these regions are identified by the learning very rapidly, requiring very few experiments to resolve. The paucity of required training points outside of the immediate regions of second-order equivalence illustrates the ability of this active learning framework to converge to a solution very efficiently. Videos showing the evolution of the boundary with each experiment can be found in the SM.

### Phase 3: Random field terraformer configuration

**Figure 3:**
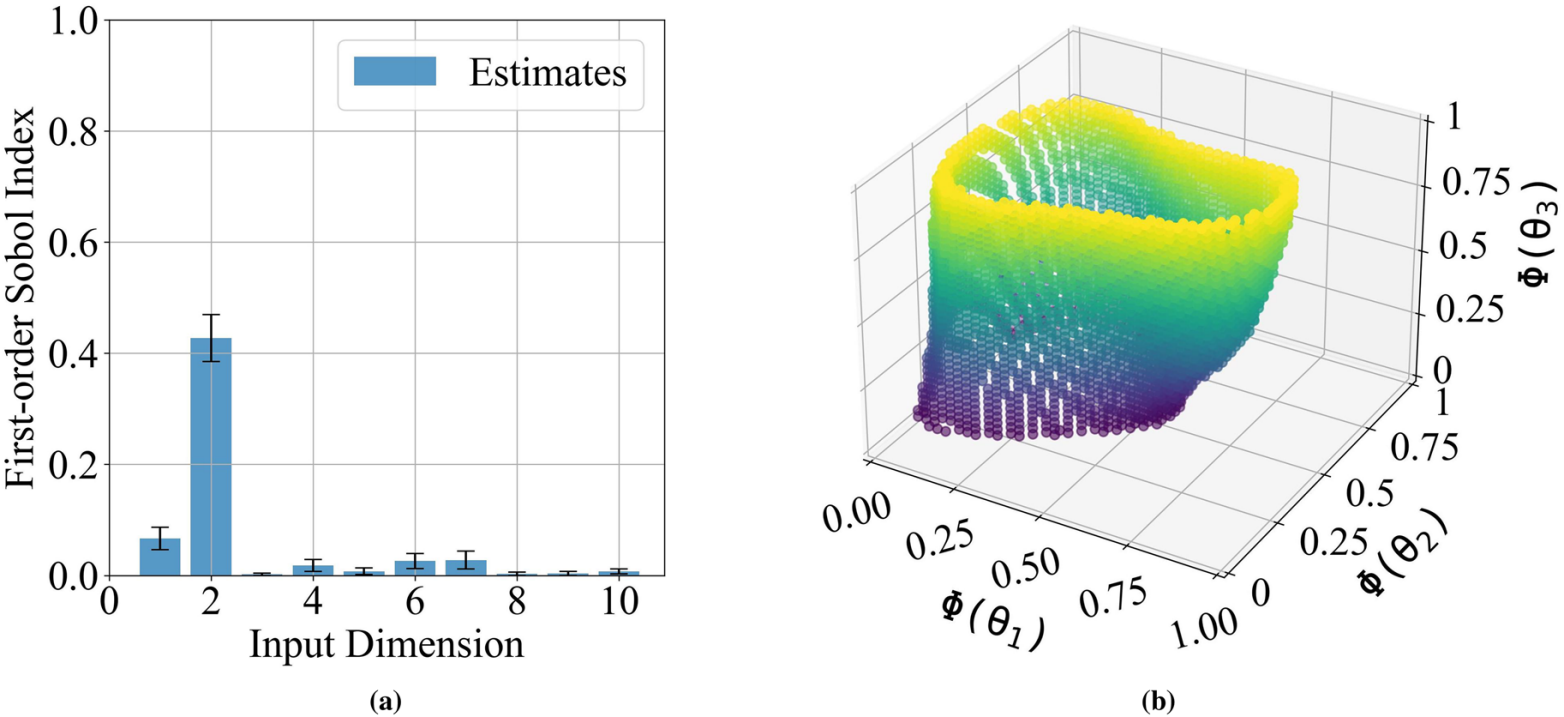
Phase 3 random field terrain study—(**a**) Main effect Sobol indices for each parameters of the KL expansion. (**b**) Second-order equivalence region in the first 3 dimensions identified from active learning. Note that axes are transformed to [0,1] by the standard normal cumulative distribution function $\Phi (\cdot )$. Points are colored according to coordinate $\theta _3$.

In the final phase, we exercised the active learning for a more complex Terraformer element configuration modeled as a one-dimensional Gaussian random field in the alongwind direction. The random field possesses the following covariance function

4 $$\begin{aligned} C(x_1,x_2) = \sigma ^2\exp {\left( -a|x_2-x_1|\right) }\cos \left( \omega |x_2-x_1|\right) \end{aligned}$$

where $x_1, x_2$ are two points along the length of the Terraformer, $\sigma ^2=100$ mm is the variance of the field, $\omega$ is the wave number, and *a* and $\omega$ are selected such that the length scale of the covariance function is given by $L=a/(a^2+\omega ^2) = 3000$ mm, or approximately 1/6 of the Terraformer length. Shorter length scales may not adequately resolve the random field due to the discrete element construction of the Terraformer, while longer length scales would appear undesirably flat over the fetch length. We then expand the Gaussian random field using the Karhunen-Loeve Expansion^37^ shown in Table 1 where $\lambda _i$ and $f_i(x)$ are the eigenvalues and eigenvectors of the covariance function respectively and $\theta _i, i=1,\ldots , n$ are standard normal random variables. We began by setting $n=10$ and ran active learning for 300 total experiments, 27 initial training experiments and 273 using active learning with the newly developed MUSIC learning function (Table 2) to compute sensitivity indices and reduce the dimension. Sensitivity estimates shown in Fig. 3a indicated that second-order equivalence is, by far, most sensitive to dimension $\theta _2$, corresponding to the second eigenvector, and has some modest sensitivity to dimension $\theta _1$. All other main effect sensitivities are very small and considered negligible. These sensitivities do not add to one, as the remaining influences are from interactions. The GP-based sensitivity method used in the MUSIC learning function can also be used to compute interaction sensitivities. In this case, nearly all interactions sensitivities are attributed to interactions with $\theta _2$ such that its total effect sensitivity (main effects plus interactions) is close to 0.9.

Using a reduced dimension expansion with only $n=3$, justified by the active learning sensitivity analysis, we performed active learning with the noisy U-function starting with 27 initial experiments drawn using stratified sampling and a total of 197 experiments over 79 h. The 3D surface in Fig. 3b shows the boundary of the predicted region of 2nd-order equivalence in this reduced 3D space with the parameters shown by their probabilities $\Phi (\theta _i)$, where $\Phi (\cdot )$ is the standard normal cumulative distribution function. This equivalence region is consistent with the results of the sensitivity analysis, showing that indeed the $\theta _2$ dimension is the most sensitive. The region is relatively narrow in this dimension, extending only over the center of the distribution. Values in the tails of the distribution result in wind turbulence profiles that are not equivalent to the uniform 80mm element height benchmark. $\theta _1$ also appears to have some sensitivity, although the surface is wider in this dimension indicating a more gradual change in the second-order difference performance function. In $\theta _1$ the region extends almost completely into the lower tail (corresponding to large negative values), but does not extend into the upper tail. Finally, the $\theta _3$ dimension has very little sensitivity, with only very small variations in the region in this dimension. The region remains entirely open in both the upper and lower tails, meaning that even extreme values of this parameter in the tails of the distribution do not change whether the turbulence profile field is equivalent.

The equivalence region in Fig. 3b was accurately resolved with less than 200 machine learning selected experiments. We did not use the experiments from sensitivity analysis in the analysis of second-order equivalence to make these two distinct studies. If we had done so, the total number of experiments would have been even fewer. A video showing the evolution of these boundary with each experiment can be found in the SM.

## Discussion

The direct impacts of this study on the field of wind engineering are the following:

- Active machine learning can be used to efficiently produce wind fields with desired properties.
- Vastly different terrains can produce wind fields with similar second-order statistical features. However, the higher-order characteristics of these fields, which govern e.g. extreme wind loads, may not be the same.
- Wide-ranging experimental investigations that have been historically intractable are now possible, which opens a new frontier of exploration for wind researchers that may translate to improved design and analysis practices for critical infrastructure.

These will be elaborated in domain appropriate publications, but the profound larger impact of these results shows the remarkable ability for active learning to enhance the rate of discovery from large-scale experiments. We first explored the potential for active learning and demonstrated rapid convergence toward the learning objective in a two dimensional parameter space. Phase 1 was notably inefficient since we explored the use of two learning functions, yet we achieved our objective with only 253 experiments and approximately 2 work weeks of automated experimental effort. By contrast, in order to achieve similar resolution of the second-order equivalence boundary using a naïve Monte Carlo or tensor product space-filling experimental design approach would require an order of magnitude increase in resources, estimated at approximately 2500 experiments and 500 h (3 months) of full-time experimental effort, with many of these experiments wasted in regions of little influence. The improvement was even more remarkable in phase 2, where the learning was conducted in less than 100 total experiments for each case, a 25-fold reduction in effort compared with an expected 2500 experiments using a standard space-filling design.

The full benefits of pairing active learning with this experimental facility were truly realized in phase 3, where a full exploration of the 10-dimensional random field Terraformer configuration would require billions of experiments. A space-filling design with an average density of 10 samples in each dimension (e.g. a tensor product design) requires exploration of $10^{10}$ total Terraformer configurations, which would require several thousand human lifetimes to complete. Even if variance reduction or information maximization approaches for experimental design were applied, tens of thousands of experiments would be required and resulting in thousands of hours of experiments, which is still prohibitively large. Instead, the active learning sensitivity analysis required only 300 experiments to reduce the dimension to a modest 3 dimensions and only approximately 200 additional experiments to learn the equivalence region. In previous wind tunnel experimental frameworks, these studies would have been inconceivable. Of course, this owes to both the active learning and the state-of-the-art automation capabilities at the University of Florida BLWT facility. This highlights what is possible, through active learning, when such capabilities are available in experimental facilities (beyond just the BLWT) and serves as a strong motivator to construct new automated experimental facilities and modernize existing facilities with these capabilites.

The impact of these findings extends far beyond the wind tunnel applications. With the active learning framework presented and demonstrated in this paper, active learning can have profound impacts on discovery across the scientific landscape. We have shown that active learning can have the same profound impact on physical experimentation and exploration as it has had on computational discoveries over the past 20 years. As a result, coupling active learning for sensitivity analysis to reduce dimension and other objectives for fundamental discovery in physical testing can transform the scientific endeavor in many areas from health to physics. That is not to say that dimension reduction of the order we achieved will always be possible, especially for problems that involve hundreds, or thousands, of parameters. But, in areas where this dimension reduction and learning are possible, the rate of scientific discovery is sure to increase substantially.

## Supplementary Information

Supplementary Information 1.

Supplementary Video 1.

Supplementary Video 2.

Supplementary Video 3.

Supplementary Video 4.

## Data Availability

The datasets generated and/or analysed during the current study are available through the DesignSafe repository^38^.
