# Supplementary material for: Active learning applied to automated physical systems increases the rate of discovery
Source: Sci Rep. 2023 May 24;13:8402. doi: 10.1038/s41598-023-35257-7 (PMC10209069; doi:10.1038/s41598-023-35257-7)
Supplement: Supplementary file 1 — Supplementary Information 1. [file 41598_2023_35257_MOESM1_ESM.pdf]

Supplemental Materials for "Active Learning Applied to Automated Physical Systems Increases the Rate of Discovery"

Methods

The following presents additional details pertaining to the application of the active learning framework to the BLWT as presented in the "Methods" section.

Quantities of Interest

The "Quantities of Interest" Section introduces the turbulence intensity profile as our QOI and the difference between turbulence intensity profiles as the measure of second-order equivalence. Here, we provide additional details defining the turbulence intensity profile, how distances between them are computed, and statistical analysis conducted to establish the threshold for second-order equivalence.

Turbulence Intensity Profiles

Under mild assumptions, wind velocity at a point in the BLWT under steady-state flow conditions is a stationary and ergodic random process. The complete joint probability distribution of this random process cannot be practically characterized from finite spatial and temporal measurements in the BLWT. Instead, quantities of interest for these wind velocity random processes are derived from the statistical moments of the process at different heights (z) above the floor. Turbulence intensity is a measure of the second-moment of the process and is a widely used measure of turbulent flow. We describe how turbulence intensity profiles are derived from wind velocity measurements next.

At each probe location having elevation z, we collect $N = 37,888$ wind velocity measurements over 30 seconds, at a sampling rate of 1262.93 Hz. Assuming that the wind velocity records are stationary and ergodic, we estimate the mean and variance of the wind velocity u at elevation z by performing a temporal averaging over each record and a spatial averaging over the three transverse probe locations at that elevation as

$$\hat{U}(z) = \sum_{i=1}^{N} \sum_{j=1}^{3} u_{ij}(z)$$

and

$$\hat{\sigma}_u^2(z) = \frac{1}{3N-1} \sum_{i=1}^{N} \sum_{j=1}^{3} (u_{ij}(z) - \hat{U}(z))^2$$

The turbulence intensity at elevation z is defined as the ratio of the standard deviation and the mean values given in (Eq. 1) of the manuscript. Performing these operations at each recorded elevation produces a vertical profile of the turbulence intensity across the region of interest (inertial sublayer).

Distances between Turbulence Intensity (TI) Profiles

Consider two turbulence intensity profiles, $l_{x1}(z)$ and $l_{x2}(z)$, evaluated at n discrete elevations z_i; $i = 1, \ldots, n$. We define the mean square distance between two profiles as:

$$d^2 = \sum_{i=1}^{n} \Delta_i^2$$

where $\Delta_i = l_{x2}(z_i) - l_{x1}(z_i)$. In the absence of noise, d provides an exact measure of distance between two turbulence intensity profiles. However, the measured profiles have several sources of noise including noise from atmospheric variations, measurement error, and statistical variations from finite velocity records, among others. By introducing noise, we can express the mean square distance as

$$d^2 = \sum_{i=1}^{n} (\Delta_i + \epsilon_i)^2$$

where ϵ_i are assumed to be independent normal random variables with zero mean and standard deviation σ_ϵ. Expanding this equation yields

$$d^2 = \sum_{i=1}^{n} (\Delta_i^2 + 2\Delta_i\epsilon_i + \epsilon_i^2)$$

The first term is the true distance. Given that ϵ_i is a normal random variable, the second term will also follow a normal distribution with $2\sum_{i=1}^{n} \Delta_i\epsilon_i \sim N(0, 4\sum_{i=1}^{n} \Delta_i^2\epsilon_i^2)$. Moreover, the third term will follow a chi-square distribution as $\sum_{i=1}^{n} \epsilon_i^2 \sim \chi^2(n\sigma_\epsilon^2, 2n\sigma_\epsilon^4)$. Next, we see that for very similar profiles in the presence of moderate noise, $\Delta_i < \epsilon_i$ and the error is dominated by the third (chi-square) term. Conversely, when $\Delta_i > \epsilon_i$ the noise is dominated by the second (normal) term. However, we note that the total contribution of these two terms to the root mean square distance d across a wide range of Δ_i is approximately constant. This can be observed directly in the attached "**Data file S1.ipynb**".

Measure of Equivalence

As previously mentioned, when Δ_i is small the error is dominated by $\sum_{i=1}^{n} \epsilon_i^2$. In fact, when $\Delta_i = 0$ the error in d^2 follows the χ^2 distribution given above. Given that ϵ_i is unknown, we can exploit this χ^2 distribution to infer a measure of statistical equivalence by performing a number of repeated identical experiments having $\Delta_i = 0$, $\forall\, i$.

We conducted 25 repeated benchmark experiments (a uniform grid with element heights all set to 80mm) across several days to account for varying atmospheric conditions. From these 25 experiments, we estimated the mean turbulence intensity profile, which we consider to be the "true" (approximately error free) profile for the benchmark terraformer configuration. Note that this turbulence intensity profile was compared with known profiles for uniform height terraformer configurations explored in reference [25] from the manuscript. From this true profile, we then compute the distance d between each of the 25 individual turbulence intensity profiles and the true profile. Recognizing that all deviation between these profiles results from sources of noise, i.e. $\Delta_i = 0$; $\forall\, i$, we fit a χ-distribution to the distances as illustrated in (Fig. S1).

Under the assumption that the noise sources do not change appreciably across the range of terraformer configurations considered, we establish statistical tests for second-order equivalence by first defining the 99% upper confidence interval of the distances, denoted d_{99} and shown in Fig. S1. Two profiles are considered statistically equivalent if their distance $d < d_{99}$. In other words, if $d > d_{99}$ we can assert with

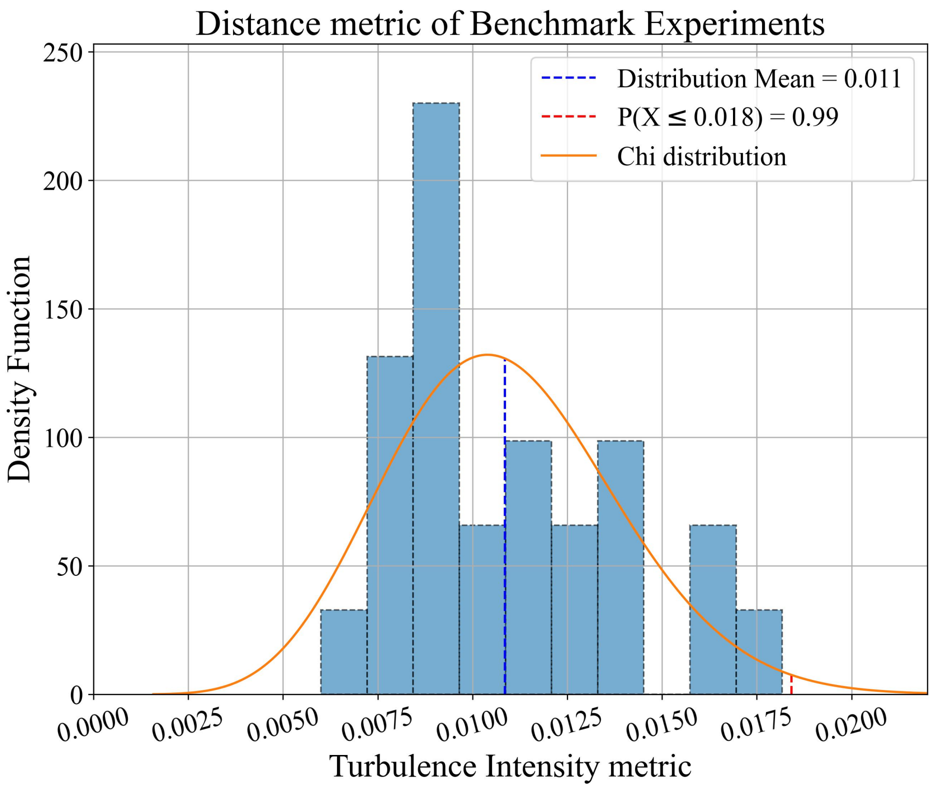

Fig S1: Distribution of distances between individual benchmark turbulence intensity profiles and the "true" benchmark turbulence intensity profile taken as the average over all individual profiles. The orange line represents the χ-distribution fit to the data.

99% confidence that (under the stated assumptions) the profiles are not second-order equivalent. Two profiles are therefore considered second-order equivalent if $d < d_{99}$ because we cannot state with a high degree of statistical confidence that they are different.

Experimental Parameterization

Across the three studies, the Terraformer terrain is configured to vary according to the following functions:

Sinusoidal Terrain (Phase 1)

For sinusoidal terrain in Phase 1, the element height H at location x along the fetch is specified by:

$$H(x) = \mu_H + A\sin(\omega(29500 - x) + \pi), \quad x \in [11200, 29500] \text{ mm}$$

where $\mu_H = 80$ mm is the mean element height, $A \sim \text{Uniform}[0, 80]$ mm is the amplitude of the sine wave, and $\omega \sim \text{Uniform}[-2\pi/3000, 2\pi/3000]$ rad/mm is the wave number. All waves are designed to have consistent 80 mm height at the end of the terraformer grid elements ($x = 29500$mm). Negative wave numbers are considered to induce a phase shift of π. Fig. S2(a) illustrates the form of the Terraformer element grids for a sinusoidal wave with parameters $\mu = 80$mm, $A = 30$mm and $\omega = -\pi/3000$rad/mm.

Sinusoidal Terrain (Phase 2a)

For sinusoidal terrain in Phase 2a, the element height H at location x along the fetch is specified by:

$$H(x) = \mu_H + A\sin\left(-\frac{\pi}{3000}(29500 - x)\right)$$

where $\mu_H \sim \text{Uniform}[50, 110]$ mm is the mean element height and $A \sim \text{Uniform}[10, 30]$ mm is the amplitude of the sine wave. This terrain configuration used here is same as study 1, except the wavenumber is fixed at $-\pi/3000$ and the effect of mean height is studied here along with the amplitude.

Triangular Wave Terrain (Phase 2b)

For triangular wave terrain in Phase 2b, the element height H at location x along the fetch is specified by:

$$H(x) = \mu_H + \frac{2A}{\pi}\arcsin\left(\sin\left(-\frac{\pi}{3000}(29500 - x)\right)\right)$$

where $\mu_H \sim \text{Uniform}[50, 110]$ mm is the mean element height and $A \sim \text{Uniform}[10, 30]$ mm is the amplitude. Fig. S2(b) illustrates the form of the Terraformer element grids for a triangular wave by converting a sinusoidal wave shown in Fig. S2(a).

Square Wave Terrain (Phase 2c)

For square wave terrain in Phase 2c, the element height H at location x along the fetch is specified by:

$$H(x) = \mu_H + \frac{2A}{\pi}\text{sgn}\left(\sin\left(-\frac{\pi}{3000}(29500 - x)\right)\right)$$

where $\mu_H \sim \text{Uniform}[50, 110]$ mm is the mean element height and $A \sim \text{Uniform}[10, 30]$ mm is the amplitude. Fig. S2(c) illustrate the form of the Terraformer element grids for a square wave by converting a sinusoidal wave shown in Fig. S2(a).

Random Field Terrain (Phase 3)

For random field terrain in Phase 3, the element height H at location x along the fetch is specified by:

$$H(x) = \mu_H + \sum_{i=1}^{d} \theta_i \sqrt{\lambda_i} f_i(29500 - x)$$

where $\mu_H = 80$ mm is the mean element height, $\theta_i \sim N(0, 1)$ are independent standard normal random variables, and λ_i, $f_i(x)$ are the eigenvalues and eigenfunctions of the following covariance function

$$C(x_1, x_2) = \exp\left(-a|x_2 - x_1|\right)\cos\left(\omega|x_2 - x_1|\right)$$

where a and ω are selected such that the length scale of the covariance function is given by $L = a/(a^2+\omega^2) =$ 3000 mm. Fig. S2(d) shows the terrain configuration for one of the initial experiments (Exp: 21, $d = 10$).

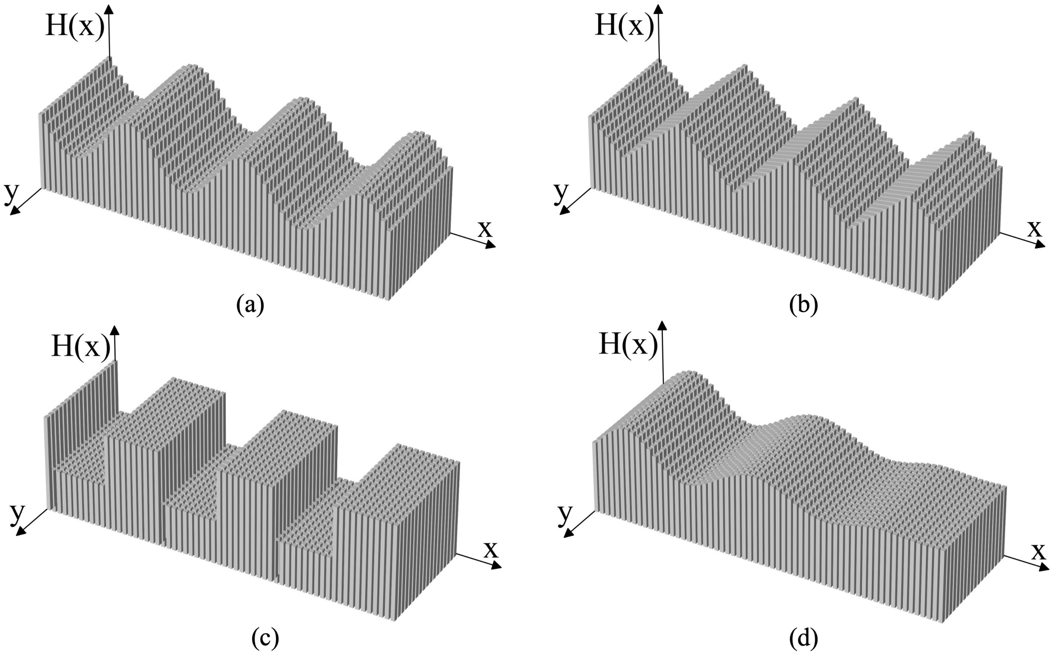

Fig S2: (a) A sinusoidal wave form with $A = 80\text{mm}$ and $\omega = -\pi/3000(\text{rad/mm})$. (b) A triangular wave form generated using same sine wave. (c) A square wave form. (d) An example of a random field terrain generated using following parameters ($d = 10$ and $\boldsymbol{\theta} = [0.48, 1.29, 1.17, -2.31, -0.14, 0.48, 1.26, 2.59, 0.49, 1.37]$).

Automated Measurement Instrumentation

Here we provide a time-lapse video of the experimental setup and data collection process at the University of Florida Boundary Layer Wind Tunnel. The video begins by setting the Terraformer, starting fans and waiting for them to achieve the steady-state wind velocity. Next, wind velocity is measured at discrete locations in a plane perpendicular to the flow using an automated multi-degree-of-freedom instrument gantry capable of traversing longitudinally, laterally, and vertically. The gantry system is equipped with three fast-response four-hole Cobra Velocity Probes to capture discrete point measurements of u, v, and w velocity components and static pressure within a $\pm 45^\circ$ acceptance cone. Upon completion of data collection, the fans are terminated and the Terraformer elements are moved to create a new terrain. The process repeats.

The video can be found in the included file **Powell Lab.mp4**.

Learning Function

The following section provides a review of the learning functions employed in this study and some discussion on how they are modified to include noise.

U-function

Introduced in [28], the U-learning function is used in the field of reliability analysis. The main objective of this function is to identify points with a high probability of incorrectly predicting the sign of output.

These points are added to the training data to improve the accuracy of the predicted limit surface; the surface where the function is equal to zero. Points selected by this algorithm are either very close to the limit surface, have high prediction uncertainty, or both.

For a normal random variable, the ratio of the absolute value of the mean and standard deviation represents the distance, measured in standard deviations, between mean value and zero. The U-learning function is defined as the ratio of the absolute value of the GP regression prediction (i.e. GP mean) and the uncertainty, (i.e. standard deviation) at a point x and can be expressed as:

$$U(x) = \frac{|\hat{y}(x)|}{\hat{\sigma}_{\hat{y}}(x)} \tag{1}$$

where $\hat{y}(x)$ is the GP surrogate prediction (mean) at x and $\hat{\sigma}_{\hat{y}}(x)$ is the standard deviation in the prediction. The function therefore yields low values for samples with predictions very close to zero or very high standard deviation and the algorithm developed in [28] selects the point that minimizes $U(x)$.

Modified U-function

The U-function can be modified to account for noise in the training data. Let us define the noise using an independent normal random variable, ϵ_d, with mean zero and standard deviation σ_{ϵ_d} such that the input-output relation follows

$$\tilde{y}(x) = y(x) + \epsilon_d$$

where $\tilde{y}(x)$ is the noisy output measured for input x and $y(x)$ is the exact output value at x. The main idea is to remove the effect of noise from the surrogate model, and compute the U-function on the noise-free prediction. Taking the variance of both sides and rearranging the equation leads to the following variance relation

$$\sigma_y^2(x) = \sigma_{\tilde{y}}^2(x) - \sigma_{\epsilon_d}^2$$

Plugging this expression into Eq. 1, the standard deviation of the actual output is expressed in terms of the measured variance and the error due to noise. This removes the uncertainty in the measured data due to noise and results in the following modified "noisy" U-function.

$$U_n(x) = \frac{|\hat{y}(x)|}{\sqrt{\sigma_{\tilde{y}}^2(x) - \sigma_{\epsilon_d}^2}^{+}} \tag{2}$$

We note that the noise standard deviation σ_{ϵ_d} is assumed to be known and is statistically estimated from the repeated benchmark experiments described above under the assumption that the noise does not depend strongly on the roughness element configuration.

Expected Improvement for Global Fit (EIGF)

The EIGF learning function, as defined in [29], is designed to search for the best set of training points to achieve a global fit with low error. The function is derived by defining an improvement function ($I(x)$) given by:

$$I(x) = (Y(x) - y(x^*))^2$$

where $Y(x)$ is unknown observation at the point x and $y(x^*)$ is the observed output at the nearest point x^* in the training data. Assuming $Y(x)$ is a normal random variable, the expected value of the improvement function is determined, as derived in [29], to be

$$E[I(x)] = (\hat{y}(x) - y(x^*))^2 + \sigma_{\hat{y}}^2(x) \tag{3}$$

where $\hat{y}(x)$ is the surrogate prediction at input x and $\sigma_{\hat{y}}(x)$ is the standard error in the prediction value. The algorithm then seeks the point that maximizes this expectation at each iteration.

Modified Expected Improvement for Global Fit (EIGF)

The EIGF learning function is modified to incorporate the noise observed in the measured data as follows. Let us again consider the noise as an independent normal random variable (ϵ_d) with zero mean and standard deviation σ_{ϵ_d}. Following a similar derivation as shown for modified U-learning function, modified "noisy" EIGF learning function can be expressed as follows:

$$E[I(x)] = (\hat{y}(x) - y(x^*))^2 + \sigma_{\hat{y}}^2(x) - \sigma_{\epsilon_d}^2 \tag{4}$$

Again, we note that the noise standard deviation σ_{ϵ_d} is assumed to be known and is statistically estimated from the repeated benchmark experiments described above under the assumption that the noise does not depend strongly on the roughness element configuration.

Minimize Uncertainty in Sensitivity Index Convergence (MUSIC)

The Sobol index for a given variable X_i is defined by its relative contribution to the total variance of the output $\mathcal{Y}(X)$, which can be expressed as:

$$S_i = \frac{\mathrm{Var}_{X_i}(E_{X_{\sim i}}[E[\mathcal{Y}(X)]|X_i])}{\mathrm{Var}(E[\mathcal{Y}(X)])}$$

To compute Sobol indices using a GP surrogate, we start by taking the expectation of the GP, $\mathcal{Y}(X,\omega)$, over all inputs except 'X_i' and we define this new quantity as the main effect GP, i.e. $A(X_i) = E[\mathcal{Y}(X,\omega)]|X_i]$. To minimize the uncertainty in Sobol index estimation, we apply the EIGF learning function to this main effect GP. This results in the following learning function, which we refer to as the MUSIC learning function,

$$♪(x_{j,i}) = w_i\left[(\mu_{A_i}(x_{j,i}) - \mu_{A_i}(x_{j^*,i}))^2 + \sigma_{A_i}^2(x_{j,i})\right]$$

where $\mu_{A_i}(.)$ and $\sigma_{A_i}(.)$ are the prediction and standard error of the main effect GP A_i. The optional weights, w_i are defined as $w_i = \mu_{S_i}/\sum_i \mu_{S_i}\ \forall\ \ i \in \{1,2,\ldots,d\}$ and can be used to force the learning function to explore those dimension with high sensitivity first. In this learning procedure, the point that maximizes $♪(x_{j,i})$ across all dimensions is selected as the point to add to the training set.

Results

The videos referenced in this section show the evolution of the region of second order equivalence for Phases 2 and 3. These studies started with a small set of training data points and the design set grows adaptively based using the iterative algorithms described above and in the main text. Each frame of the videos included here show the current training data set and color maps of surrogate prediction at each iteration. The next sample selected from the learning function is illustrated by a star ($\star$). The end results are shown as Fig. 2 & 3b in the manuscript.

These videos can be found in the attached files **Phase2a.mp4**, **Phase2b.mp4**, **Phase2c.mp4**, and **Phase3.mp4**.
